# Pharmacokinetics of (6S)-5-Methyltetrahydrofolate dicholine salt compared to folic acid: a randomized double-blind single dose cross-over study

**DOI:** 10.29219/fnr.v69.12633

**Published:** 2025-09-24

**Authors:** Christiane Schön, Antje Micka, Daniel Menzel, Manfred Wilhelm, Rima Obeid

**Affiliations:** 1 BioTeSys GmbH, Esslingen, Germany; 2 Department of Mathematics, Natural and Economic Sciences, Ulm University of Applied Sciences, Ulm, Germany; 3 Department of Clinical Chemistry and Laboratory Medicine, University Hospital of the Saarland, Homburg, Germany

**Keywords:** Optifolin+®, (6S)-5-MethylTHF-2-choline, (6S)-5-MethylTHF, folic acid, bioavailability

## Abstract

**Background:**

(6S)-5-Methyltetrahydrofolate ((6S)-5-MethylTHF) is the physiological folate form in biological fluids. Salts of (6S)-5-MethylTHF may have advantages compared to folic acid and are increasingly used in foods and supplements.

**Objective and design:**

The present study describes the physicochemical properties of the (6S)-5-MethylTHF-2Chol salt as a source of methylfolate with respect to solubility, conductivity, and melting point. The pharmacokinetics of (6S)-5-MethylTHF-2Chol and folic acid were compared in a randomized controlled double-blind cross-over study using a single equimolar oral dose of each of the folate substances.

**Results:**

The solubility of the dicholine salt was very high (650 mg/mL in H_2_O and 40 mg/mL in H_2_O under acidic conditions). The incremental area under the curve (iAUC_0-8h_) was significantly higher after the administration of (6S)-5-MethylTHF-2Chol compared to folic acid ([1.64-fold, *P* < 0.0001] for total folate and 2.56-fold higher for (6S)-5-MethylTHF [*P* < 0.0001]).

**Discussion and conclusions:**

The bioavailability of (6S)-5-MethylTHF-2Chol is higher compared to folic acid. The crystalline structure of (6S)-5-MethylTHF-2Chol and its water solubility are advantageous in terms of stability in nutraceutical products and absorption in the gut. (6S)-5-MethylTHF-2Chol is the source of folate that may enable the development of new applications.

## Popular scientific summary

- This study compares the bioavailability of (6S)-5-MethylTHF-2Chol and folic acid, following a single equimolar oral dose of each form in healthy subjects.
- Results demonstrate a superior bioavailability of (6S)-5-MethylTHF-2Chol compared to folic acid.
- The water solubility and the crystalline structure of (6S)-5-MethylTHF-2Chol combine positive and attractive aspects in terms of absorption and stability compared to known folate salts.
- (6S)-5-MethylTHF-2Chol is a source of folate that enables the development of new applications.

Folate is an essential vitamin that cannot be produced by the body. Vegetables, nuts, liver, and legumes are main dietary sources of folate for humans. The natural folates are water soluble and sensitive to heat, light, and acidic pH (1). Heating, washing, and long-term storage of foods may destroy up to 30–50% of their folate content. Besides that, food folates have polyglutamate chains that have to be enzymatically hydrolyzed to a single glutamate prior to intestinal absorption (2, 3). The recommended intake of folate for adults is 400 μg dietary folate equivalents (DFEs) per day according to the United States National Academy of Medicine (4). There is a high grade evidence that higher folate intake (5) and higher blood concentrations (6) are associated with a lower risk of some birth anomalies. Therefore, young women planning pregnancy and pregnant women in the first trimester are recommended to achieve an additional intake of 400–800 μg folic acid through food supplements (7). In addition, a sufficient plasma folate status contributes to normal neurological and vascular function, for example, in brain and eye.

Folic acid is a synthetic provitamin form that is traditionally added to foods and food supplements because of its stability compared to food folate. Mandatory fortification of staple foods with folic acid has been introduced in 1998 in the US and Canada with the aim of eliminating folate deficiency and increasing folate status, specifically in young women, to reduce the risk of neural tube defects in the offspring. In countries with mandatory fortification with folic acid, the population’s mean serum folate value exceeds 25 nmol/L (NHANES 2005–2006) (8). In Europe (9), where no mandatory fortification with folic acid is in place, the population mean serum folate (in the absence of supplementation) is roughly 50% lower than the US or Canadian populations (8, 10).

The bioavailability of folic acid is approximately 1.7-fold higher than that of food folate (thus 1 μg of folic acid provides 1.7 μg DFE) (11). Folic acid is absorbed and converted to dihydrofolate and tetrahydrofolate by the enzyme dihydrofolate reductase (DHFR). The capacity of DHFR to reduce folic acid may be limited at higher dosages of folic acid. Detectable concentrations of unmetabolized folic acid (UMFA) are found in serum when the oral dose of folic acid exceeds 200 μg (12). Moreover, a polymorphism in DHFR has shown associations with the risk of neural tube defects, and because folic acid has to be reduced through this enzyme, it has been suggested that reduced folate forms could have advantages over folic acid (13)

(6S)-5-MethylTHF salts are reduced folate forms and a direct source of methylfolate (14). They have a fast absorption and higher bioavailability compared to folic acid (15). The use of (6S)-5-MethylTHF salts in supplements and foods, including infant formulas and antenatal supplements, is becoming more popular (16). In addition, (6S)-5-MethylTHF constitutes the physiological dominant folate form in biological fluids, such as serum, breastmilk, and cord blood (17–19). There are several commercially available (6S)-5-MethylTHF salts, such as (6S)-5-MethylTHF-Na, (6S)-5-MethylTHF-Ca, and (6S)-5-MethylTHF glucosamine salts (20, 21), which differ in their physicochemical properties and possibilities for use in nutraceutical and pharmaceutical products and fortified foods. Based on this background, a new salt of (6S)-5-MethylTHF was developed that constitutes two choline molecules ((6S)-5-MethylTHF-2Chol). Choline is an essential nutrient and a key methyl donor that contributes to normal metabolism of homocysteine, folate, and phospholipids (22).

In this study, we report the physicochemical properties of (6S)-5-MethylTHF-2Chol and present results of a randomized controlled double-blind cross-over study to compare the pharmacokinetics of (6S)-5-MethylTHF-2Chol and folic acid following a single equimolar oral dose of each folate form.

## Present investigation

### Methods

#### Experimental conditions for the assessment of physicochemical properties

(6S)-5-MethylTHF-2Chol (Optifolin+®) was provided by the Sponsor, Aprofol AG, Appenzell, Switzerland. (6RS)-5-MethylTHF-Ca was purchased at Cerbios SA. For the solubility test, each sample of the (6S)-5-MethylTHF salt was added to purified water at pH = 6.5 and 20–25°C with stirring until complete dissolution was visible. The solubility was measured in triplicate for the 2Chol and Ca folate salts. An additional solubility test was performed in simulated gastric medium (pH = 3 and pH < 2 in water) for (6S)-5-MethylTHF-2Chol according to OECD Test No. 105 (Water solubility) (OECD, 1995) (23). The concentrations of (6S)-5-MethylTHF in water (pH 6.5) or in the simulated gastric medium (pH = 3 and pH < 2) were measured by HPLC at Passafaro’s Analytic Lab (Thayngen, Switzerland) according to the USP monograph for (6S)-5-MethylTHF-Ca.

A conductivity test was performed using an aqueous solution containing 1.5 mmol/L (0.98 mg/mL) (6S)-5-MethylTHF-2Chol or an equimolar amount of (6RS)-5-MethylTHF-Ca. The conductivity of aqueous solutions of (6S)-5-MethylTHF-2Chol or (6S)-5-MethylTHF-Ca was measured (in μS/cm) at two different concentrations: 14.8 mmol/L (or 9.8 mg/mL) and 149.6 mmol/L (or 99.6 mg/mL) at 23–25°C using a FiveGo Portable F3 conductivity meter from Mettler Toledo, Gießen, Germany. The melting point of (6S)-5-MethylTHF-2Chol has been determined by differential scanning calorimetry Mettler Toledo, STAR SW 9.01, Gießen, Germany.

For further synonyms of the different folate salts, see Supplementary file (Table S3).

#### Study design and settings

This randomized, double-blind single dose cross-over study was performed between August 2023 and November 2023 at BioTeSys GmbH, Esslingen, Germany. The study is registered at DRKS (DRKS00032494).

Eligible participants were healthy adults, aged 18–60 years, and a body mass index (BMI) between 19.0 and 30.0 kg/m^2^. The main exclusion criteria were low hemoglobin levels (defined as Hb < 12 g/dL for women or Hb < 13.0 g/dL for men), smoking, use of folate supplements within the last 3 months prior to the study or during the study, plasma homocysteine concentrations ≥ 18.0 μmol/L, blood liver enzymes > 3 fold above the upper limit of the reference range, fasting plasma glucose > 126 mg/dL, eGFR (GFR-EPI) < 60 ml/min/1.73 m^2^, history of severe medical disorders that may affect folate bioavailability, a significant cardiovascular event within the last 3 months, gastrointestinal diseases or intake of medication or dietary supplements (in the last 2 months) with impact on folate absorption and metabolism possibly interfering with this study. We also excluded people who have used antibiotics within the last 4 weeks and those regularly using non-steroidal anti-inflammatory drugs (i.e. ibuprofen and diclofenac) or aspirin. Other exclusion criteria included pregnancy and lactation, blood donation within 1 month prior to study start or during the study, or participation in another clinical study within the last 4 weeks.

The clinical study was approved by the ethics committee of Landesärztekammer Baden-Württemberg (Approval Number: F-2023-087). This study was conducted in accordance with the guidelines for Good Clinical Practice (GCP) set forth by the International Council for Harmonisation of Technical Requirements for Pharmaceuticals for Human Use (ICH), and with ethical principles based on Declaration of Helsinki. All participants provided written informed consent prior to any study specific assessments.

Study capsules contained 906 nmol (400 μg) folic acid (from Euro OTC Pharma GmbH, Germany) or an equimolar amount (603 μg) of (6S)-5-MethylTHF-2Chol (Aprofol AG, Switzerland) in addition to microcrystalline cellulose that was used as excipient. The products were provided in hard gelatine capsules as single dose after at least 10 h of fasting. The capsules were produced and randomized at the Pharmacy Roter Ochsen AG, Schaffhausen, Switzerland. Randomization was stratified for sex, and a block size of *n* = 4 was used. Products were directly labeled according to the randomization list with participant number and visit. Participants were chronologically allocated at their first kinetic day. Both the investigators and the participants were blinded for the content of the capsules. The folate content of the study capsules was verified by HPLC according to the USP monograph for (6S)-5-MethylTHF-Ca at Passafaro’s Analytic Lab (Thayngen, Switzerland).

The inclusion and exclusion criteria were verified during the screening visit at the study site. Participants who met the study criteria were randomized to receive either folic acid or an equimolar amount of (6S)-5-MethylTHF-2Chol. On the kinetic day at the study site, the study product (a single capsule containing 906 nmol folate) was provided with 150 mL water under supervision of the study personnel. After a washout period of 14 days, participants returned to the study site and received the second substance in the same way. All subjects were asked to maintain their regular lifestyles and usual extent of physical activities during the whole study period.

Venous blood samples were collected into Ethylenediaminetetraacetic acid (EDTA)-tubes immediately before the intake of study products (after an overnight fast) and 15 min, 30 min, 1 h, 1.5 h, 2 h, 3 h, 4 h, 6 h, 8 h, and 24 h thereafter. The EDTA blood was centrifuged within 30 min (for 10 min at 3,000 x g at 4°C), and the EDTA-plasma was separated and stored at <-70°C until analysis.

Subjects received standardized meals for 24 h after intake of the study products. One hour and three hours after the folate dose, a folate poor drink (Calshake Vanilla, Fresenius) with lactose-free milk was provided. Folate content was verified in the drink (0.44 μg/100 mL) by VitaFast^®^ microbiological technology for foods at SGS Institut Fresenius, Berlin, Germany. Thus, the drink provided 2.8 μg additional intake of folate that is considered as negligible compared to the study dose. Further folate poor meals were served at the study site 6, 8, and 12 h after the oral dose.

#### Blood analyses and analytical methods

The concentrations of plasma total folate were measured at all time points using automated electrochemiluminescence binding assay on Cobas system (Elecsys, Roche) at the Saarland University Hospital. The plasma concentrations of (6S)-5-MethylTHF, UMFA, and non-methylTHF were measured using UPLC-MS/MS at the Saarland University Hospital according to the method of Kirsch et al. (24).

#### Sample size estimation and statistical analyses

Sample size estimation was performed a priori based on a previous bioavailability study on a sodium folate salt (21), indicating an effect size for incremental area under the curve (iAUC) of 0.98. With consideration of power of 80% and a significance level of 5%, a sample size of *n* = 11 based on plasma total folate was calculated for the cross-over design. As data were planned to be evaluated stratified according to sex and equally sized sequence groups, the sample size was set to 24 subjects (12 women and 12 men). The pharmacokinetic endpoints iAUC, ∆C_max_, and T_max_ were determined from individual concentration time curves using the trapezoidal method. iAUC was determined over two time periods of 0–8 h and 0–24 h. The pharmacokinetic endpoints of total folate and (6S)-5-MethylTHF were compared between (6S)-5-MethylTHF-2Chol (test product) and folic acid (reference product).

iAUC and ∆C_max_ based on log-transformed data were analyzed using a linear mixed model taking into account the kinetic day (2 levels), sequence of the products (2 levels), the study product (2 levels), and baseline folate concentration within each study period as fixed effects and subject as random effect. In addition, the geometric mean ratio with 90% confidence interval (CI) of iAUC and ∆C_max_ for total folate and (6S)-5-MethylTHF between (6S)-5-MethylTHF-2Chol and folic acid was determined from the linear mixed models.

The difference in T_max_ between (6S)-5-MethylTHF-2Chol and folic acid was evaluated with non-parametric test statistics under consideration of the cross-over design.

## Results

### Physical and chemical properties

The crystalline (6S)-5-MethylTHF-2Chol dissolves in pure water at pH 6.5 at 650 mg/mL. In simulated gastric juice, the salt dissolved at pH 3 and pH < 2, with a concentration of 40 mg/mL readily. The aqueous equimolar solution of (6S)-5-MethylTHF-2Chol and (6RS)-5-MethylTHF-Ca (1.5 mmol/L) showed a conductivity of 200–225 μS/cm. Hence, both organic (6S)-5-MethylTHF salts form ions with the same degree of ionization or dissociation when dissolved in water. The conductivity of (6S)-5-MethylTHF-2Chol in aqueous solution at a concentration of 14.8 mmol/L (9.8 mg/mL) and 149.6 mmol/L (99.6 mg/mL) was 1,568 μS/cm and 9,659 μS/cm, respectively. (6S)-5-MethylTHF-2Chol is a crystalline salt that melts at 230°C with subsequent decomposition.

### Bioavailability of (6S)-5-MethylTHF-2Chol compared to folic acid

**Fig. 1:**
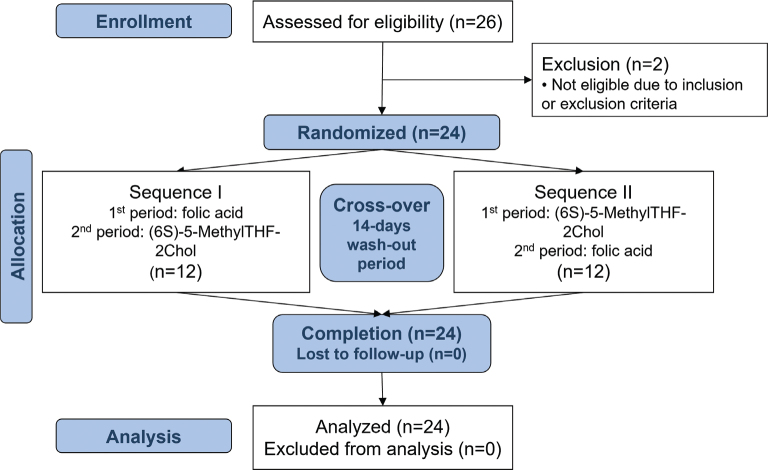
Study flow diagram.

Twenty-four participants (50% men, 50% women), who met the study inclusion criteria and none of the exclusion criteria, were enrolled in the study and completed the study in its entirety (Fig. 1). The key characteristics of the 24 participants are shown in Table 1.

**Table 1:** Characteristics of study participants at the screening visit (*n* = 24)

Variable	Mean (95% CI)
Age [years]	34.6 (29.3−40.0)
BMI [kg/m^2^]	23.0 (21.6−24.4)
Systolic blood pressure [mmHg]	123 (118−128)
Diastolic blood pressure [mmHg]	84 (80−88)
Hemoglobin [g/dL]	14.3 (13.8−14.7)
Plasma homocysteine [μmol/L]	7.3 (6.5−8.0)

**Fig. 2:**
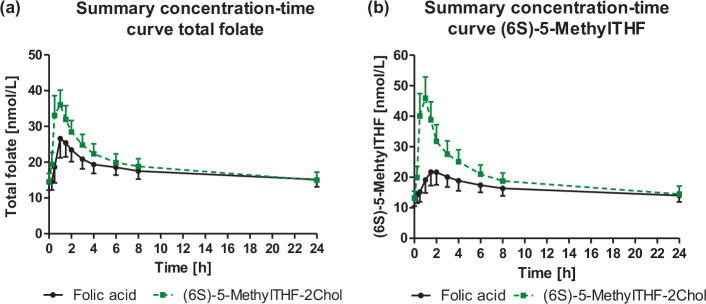
Response of plasma concentrations of (a) total folate [nmol/L] and (b) (6S)-5-MethylTHF [nmol/L] after the intake of the study products (data are shown as mean ± 95% CI).

Baseline concentrations of total folate and (6S)-5-MethylTHF were comparable between the two assessment days (*P* = 0.9672 and *P* = 0.9146 for within-individual comparisons, respectively, Supplementary Table S1). After intake of (6S)-5-MethylTHF-2Chol, plasma concentrations of total folate and (6S)-5-MethylTHF increased compared to baseline concentrations after 15 min of the oral folate intake, and this raise was maintained for 8 h (Fig. 2). After the folic acid intake, a significant increase in plasma concentrations of total folate compared to baseline concentrations was observed after 30 min and lasted until 6 h after the oral dose. Plasma concentrations of (6S)-5-MethylTHF showed a significant increase after 1 h of administering folic acid, and the concentrations remained higher than baseline concentrations up to 8 h after the oral dose. The plasma concentrations of (6S)-5-MethylTHF after (6S)-5-MethylTHF-2Chol (measured by Liquid Chromatography – Tandem Mass Spectrometry (LCMS-MS) and isotope internal standard) were higher than those of total folate (electrochemiluminescence binding assay), which may be due to different assay principles, see Supplementary Table S1. The LCMS-MS method measures the folate form selectively, whereas the immune binding assay could be influenced by the affinity of folates to the assay binding protein.

**Fig. 3:**
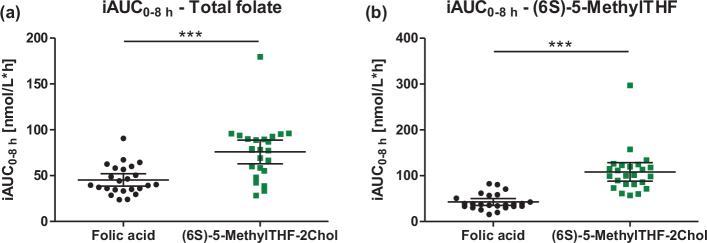
Distribution of iAUC_0-8h_ for (a) plasma total folate [nmol/L*h] and (b) plasma (6S)-5-MethylTHF [nmol/L*h] after the intake of study products; data are shown as mean ± 95% CI; between group ***P < 0.0001.

The iAUC_0-8h_ of plasma concentrations of total folate was significantly higher after the intake of (6S)-5-MethylTHF-2Chol in comparison to after the intake of folic acid (1.64-fold, between group *P* < 0.0001, Fig. 3 and Table 2). The iAUC_0-8h_ for plasma (6S)-5-MethylTHF was higher for (6S)-5-MethylTHF-2Chol in comparison to that after folic acid (2.56-fold, *P* < 0.0001). A larger iAUC of plasma concentrations of total folate and (6S)-5-MethylTHF after (6S)-5-MethylTHF-2Chol compared to that after folic acid was observed in the observation periods 0–8 h and 0–24 h. In line with this, ∆C_max_ was significantly higher after (6S)-5-MethylTHF-2Chol than after folic acid (Table 2). Time to reach maximum folate concentrations (T_max_) was reached faster after the intake of (6S)-5-MethylTHF-2Chol than after folic acid (for plasma total folate: T_max_ = 0.8 h vs. 1.6 h (mean), between group *P* = 0.0020; for plasma (6S)-5-MethylTHF: T_max_ = 0.8 h vs. 2.8 h (mean), between group *P* < 0.0001, respectively). The higher bioavailability after the intake of (6S)-5-MethylTHF-2Chol in comparison to folic acid is also confirmed in both subgroups of women and men with exception of iAUC_0-24h_ for total folate in women, see Table 2. A significant rise of UMFA concentration was observed after the intake of folic acid (∆C_max_: 1.88 ± 1.81 nmol/L [mean ± standard deviation {SD}]). The median T_max_ for UMFA was 1h (25th–75th percentile: 1.0–1.5 h). The concentrations of UMFA did not change in the 8 h after the intake of (6S)-5-MethylTHF-2Chol (∆C_max_ 0.06 ± 0.12 nmol/L [mean ± SD]). Plasma concentrations of non-MethylTHF forms (i.e. (6R)-5,10-MethenylTHF and (6S)-5-FormylTHF) were low and below the assay detection limit in the majority of the samples with no systematic increase after the intake of the products (data not shown).

**Table 2:** Pharmacokinetic parameters (iAUC_0-8h_, iAUC_0-24h_, and ∆C_max_) of plasma total folate and (6S)-5-MethylTHF after single folate dose

Pharmacokinetic endpoint	Total folate				(6S)-5-MethylTHF			
	
	Geometric mean Folic acid	Geometric mean (6S)-5-MethylTHF-2Chol	Geometric mean ratio (90 % CI)	*P* *	Geometric mean Folic acid	Geometric mean (6S)-5-MethylTHF-2Chol	Geometric mean ratio (90 % CI)	*P* *
iAUC_0-8h_ [nmol/L*h](*n* = 24)	42.8	70.2	1.64 (1.41–1.90)	< 0.0001	39.5	101.2	2.56 (2.15–3.05)	< 0.0001
iAUC_0-8h_ men (*n* = 12)	40.0	80.9	2.02 (1.65–2.48)	< 0.0001	32.6	100.5	3.08 (2.33–4.07)	< 0.0001
iAUC_0-8h_ women (*n* = 12)	45.8	61.1	1.33 (1.08–1.65)	0.0342	47.8	102.2	2.14 (1.79–2.55)	< 0.0001
iAUC_0-24h_ [nmol/L*h](*n* = 24)	73.9	108.0	1.46 (1.20–1.78)	0.0033	71.4	152.5	2.14 (1.67–2.73)	< 0.0001
iAUC_0-24h_ men (*n* = 12)	69.2	139.6	2.02 (1.62–2.51)	0.0001	52.1	152.9	2.93 (2.01–4.29)	0.0001
iAUC_0-24h_ women (*n* = 12)	77.6	85.1	1.10 (0.81–1.49)	0.5890	94.9	156.5	1.65 (1.42–1.91)	0.0003
∆C_max_ [nmol/L] (*n* = 24)	12.0	22.7	1.90 (1.58–2.27)	< 0.0001	9.7	33.1	3.41 (2.91–4.00)	< 0.0001
∆C_max_ men (*n* = 12)	11.1	24.0	2.15 (1.63–2.84)	0.0001	8.6	32.4	3.79 (3.05–4.70)	< 0.0001
∆C_max_women (*n* = 12)	13.2	21.0	1.59 (1.28–1.98)	0.0032	11.2	33.4	2.98 (2.39–3.71)	< 0.0001

* *P*-values between the product groups are derived from linear mixed models.

### Safety and tolerability

Adverse events were monitored throughout the study. Between the screening and the study end, 12 adverse events were reported by 10 subjects. During the 24 h observation period, three adverse events were reported by two subjects (2x circulatory problems; due to long fasting and blood donation but resolved within a few minutes). In addition, one participant reported abdominal pain of minor intensity in the evening of a study day, but pain resolved in the next morning. None of these events was judged to be caused by the study products. No serious adverse event (SAE) occurred during the study. Blood routine parameters were checked at baseline and 24 h post-dosing. No changes were identified, which might indicate a relation to administration of the study products. Overall, tolerability of the study supplements was very good.

## Discussion

The objective of the present study was to compare the bioavailability of a new dicholine salt of (6S)-5-MethylTHF with that of folic acid in healthy adults. The findings demonstrated that folate bioavailability as assessed by iAUC_0-8h_ was 1.64-fold higher for plasma total folate and 2.56-fold higher for plasma (6S)-5-MethylTHF following administration of (6S)-5-MethylTHF-2Chol compared to folic acid. These results were further corroborated by pharmacokinetic parameters, including iAUC_0-24h_ and ∆C_max_, for plasma total folate and (6S)-5-MethylTHF concentrations.

The (6S)-5-MethylTHF and choline resulting after dissociation of (6S)-5-MethylTHF-2Chol are likely to be absorbed in the intestine. A rapid absorption of (6S)-5-MethylTHF-2Chol as indicated by reaching a maximal plasma concentrations of folate within a shorter time than after folic acid (T_max_) may be supported by the superior aqueous solubility and dissolution properties of (6S)-5-MethylTHF-2Chol compared to folic acid (Supplemental Table S2). Folic acid has a low solubility of approximately 0.01 mg/mL (25) and is almost insoluble at pH < 5 such as in the stomach milieu (26). Moreover, after folic acid is absorbed, it is enzymatically converted to (6S)-5-MethylTHF, which is also a time-limiting step. In contrast to folic acid, an aqueous solution of (6S)-5-MethylTHF-2Chol has been reported to penetrate well into the skin, which could be explained by its higher solubility in aqueous solutions (27).

The results of the present study (geometric mean ratio of AUCs of plasma (6S)-5-MethylTHF for (6S)-5-MethylTHF-2Chol to folic acid = 2.56, 90% CI: 2.15–3.05) are in line with previous single dose bioavailability studies using other (6S)-5-MethylTHF salts compared to folic acid (21, 28). In a study in women with reduced MTHFR activity, a higher AUC was observed after a single dose intake of (6S)-5-MethylTHF-Ca compared to an equimolar amount of folic acid (416 μg vs. 400 μg) (28). The geometric mean ratio of AUCs of plasma (6S)-5-MethylTHF concentrations for (6S)-5-MethylTHF-Ca to folic acid was 2.29 (90% CI: 2.03–2.55) (21, 28). Similar geometric mean ratio of AUCs for (6S)-5-MethylTHF-Na salt to folic acid was reported 2.52 (90% CI: 2.18–2.86) (21). In contrast, Pentieva et al. demonstrated equivalent bioavailability of a single dose of (6S)-5-MethylTHF-Ca and folic acid using a Lactobacillus casei microbiological assay (29). Pentieva et al. used a folic acid presaturation model (administration of 5 mg/day folic acid for 1 week, followed by 2 days free of folic acid) prior to the single oral dose, which could reduce folate bioavailability due to high baseline folate.

Tissue folate uptake takes place via reduced folate carrier (RFC) that has higher affinity for reduced folates than for folic acid (30, 31) and folate receptors, which have higher affinity for folic acid than for reduced folates. It might be argued that lower plasma concentrations of (6S)-5-MethylTHF or total folate after folic acid compared to (6S)-5-MethylTHF-2Chol could be due to faster tissue uptake kidney clearance of folic acid. However, repeated-dose supplementation with ≥ 400 μg/day reduced folate ((6S)-5-MethylTHF-Ca) induces a greater increase in red blood cell (RBC)-folate concentrations compared to folic acid (32–35), which does not support the hypothesis of faster tissue uptake of folic acid as an explanation for lower plasma folate after folic acid. In general, because the ratios of AUCs for the three (6S)-5-MethylTHF salts (dicholine, Ca, and Na) to folic acid are similar across the studies, it is expected that the bioavailability of (6S)-5-MethylTHF-2Chol on repeated-dose studies is higher than that of folic acid and similar to that of other (6S)-5-MethylTHF salts.

Mandatory fortification of foods with folic acid is not applied in Germany. This is in line with very low or undetectable concentrations of UMFA in plasma of our participants at baseline. A single dose of 400 μg folic acid, but not 603 μg (6S)-5-MethylTHF-2Chol, caused UMFA concentrations to raise in plasma, which is in line with the previously reported threshold of folic acid intake of 200 μg (12, 36). Detectable concentrations of UMFA have been reported in the majority of samples from people exposed to mandatory fortification with folic acid (17). Excessive folic acid supply during pregnancy alters cortical neurodevelopment in mouse offspring (37). However, the health implications of circulating UMFA remain unclear (38–40). For example, exposure to high dose folic acid in pregnant animals caused pseudo-deficiency of the 5,10-Methylenetetrahydrofolate reductase (MTHFR) (41) and restricted fetal development (42). One of the hypothesized mechanism is that high dose folic acid is not fully reduced by DHFR (43) and, thus, cannot be efficiently transferred to reduced folate that can be used by the fetus. Alternatively, folic acid may block the MTHFR or DHFR enzymes and impact their functions (41). Antenatal supplements commonly provide 400 to 800 μg folate, suggesting that women in countries applying mandatory fortification with folic acid may exceed the Upper Tolerable Level (UL) (1 mg folate per day) (44, 45). Over 25% of prenatal products on the US market contain a (6S)-5-MethylTHF salt as a sole source of folate or in combination with folic acid (16). Using (6S)-5-MethylTHF salts may have practical implications on the efficiency of achieving optimal blood folate concentrations at an earlier time point than when using folic acid as a sole source of folate. This, in turn, may impact the risk reduction potential of neural tube defects, especially in women with MTHFR genotype or those who start folate supplementation after conception. However, no studies exist so far to show that using (6S)-5-MethylTHF salts in pregnant women can reduce the risk of neural tube defects.

## Conclusions

In summary, this study demonstrated superior bioavailability of (6S)-5-MethylTHF-2Chol (as a direct source of (6S)-5-MethylTHF) compared to folic acid, which seems to be in a similar magnitude compared to other folate salts, for example (6S)-5-MethylTHF-Na and (6S)-5-MethylTHF-Ca. The components of (6S)-5-MethylTHF-2Chol, (6S)-5-MethylTHF, and choline, are essential in the one carbon metabolism. The water solubility and the crystalline structure of (6S)-5-MethylTHF-2Chol combine positive aspects in terms of absorption and stability compared to known folate salts. (6S)-5-MethylTHF-2Chol is seen as an alternative source of folate that could enable the development of new applications.

## Supplementary Material


