# Supplementary material for: Pharmacokinetics of (6S)-5-Methyltetrahydrofolate dicholine salt compared to folic acid: a randomized double-blind single dose cross-over study
Source: Food Nutr Res. 2025 Sep 24;69:10.29219/fnr.v69.12633. doi: 10.29219/fnr.v69.12633 (PMC12499688; doi:10.29219/fnr.v69.12633)
Supplement: Supplementary file 1 [file FNR-69-12633-s1.pdf]

Pharmacokinetics of (6S)-5-Methyltetrahydrofolate dicholine salt compared to folic acid: a randomized double-blind single dose cross-over study

Christiane Schön [1,*], Antje Micka [1], Daniel Menzel [1], Manfred Wilhelm [2] and Rima Obeid [3]

Supplementary Table S1. Mean and SD of plasma concentration of total folate and (6S)-5-MethylTHF after single oral dose of folic acid or (6S)-5-MethylTHF-2Chol

	Plasma total folate concentrations (nmol/L)				Plasma (6S)-5-MethylTHF concentrations (nmol/L)			
	**Folic acid n = 24**		**(6S)-5-MethylTHF-2Chol n = 24**		**Folic acid n = 24**		**(6S)-5-MethylTHF-2Chol n = 24**	
time [h]	Mean	SD	Mean	SD	Mean	SD	Mean	SD
0	14.4	5.3	14.5	5.7	13.1	6.0	13.2	5.3
0.25	14.6	5.6	19.4	8.0	14.3	6.5	19.9	8.5
0.5	18.7	10.4	33.1	13.1	15.2	7.6	40.1	17.3
1	26.6	12.7	36.1	9.6	19.1	10.1	45.9	16.5
1.5	25.4	9.2	31.9	9.2	21.7	10.5	38.8	14.0
2	23.4	7.8	28.4	7.6	21.7	9.8	31.8	12.8
3	20.8	6.3	24.8	7.0	20.1	7.6	27.6	10.3
4	19.4	5.9	22.3	6.7	18.9	7.8	25.2	9.1
6	18.6	5.2	19.9	5.7	17.4	5.6	21.0	7.1
8	17.5	5.3	18.8	5.1	16.4	5.8	18.8	6.2
24	15.1	4.8	15.0	5.4	14.0	5.0	14.5	6.3

Supplementary Table S2. Characteristics of (6S)-5-MethylTHF-2Chol compared to other folate forms

**Property**	**Folic acid**	**(6S)-5-MethylTHF-Calcium**	**(6S)-5-MethylTHF-Glucosamine**	**(6S)-5-MethylTHF-2Chol**
Solid structure	crystalline	crystalline	crystalline or amorphous	crystalline
Solubility in H_2O (H_2O/ pH 7)	0.01 mg/mL [1]	10 mg/mL [2]	~1000 mg/mL [3]	650 mg/mL
(H_2O/ pH 3)	insoluble	n/a	n/a	40 mg/mL*
Folate form	Oxidized form	Reduced form	Reduced form	Reduced form
Stability (solid, dry)	good	good (cryst.)/ fair (amorph)	fair (amorph)	good (cryst.)

*tested concentration (higher concentrations not tested).

Table S3: Abbreviations and synonyms of folate salts

The following Names and synonyms are used in this manuscript:
(6S)-5-MethylTHF = (6S)-5-Methyltetrahydrofolic acid, alternative names: L-Methylfolate or L-5- Methylfolate
(6S)-5-MethylTHF-2Chol = Dicholine (6S)-5-Methyltetrahydrofolate salt, alternative name: L-MTHF di Choline, L-Methylfolate 2Chol, Optifolin+®
(6S)-5-MethylTHF-Ca = Calcium (6S)-5-Methytetrahydrofolate salt, alternative name: Calcium L- Methylfolate
(6S)-5-MethylTHF-Na = Sodium (6S)-5-Methytetrahydrofolate salt, alternative name: Sodium L-Methylfolate
(6RS)-5-MethylTHF-Ca= Calcium racemic 5-Methyltetraydrofolate, alternative name: Calcium D/L-Methylfolate
Folic acid = Pteroylmonoglutamic acid

References

1. Bottari, E.; D'Ambrosio, A.; Tommaso, G. de; Festa, M.R.; Iuliano, M.; Meschino, M. Solubility of folic acid and protonation of folate in NaCl at different concentrations, even in physiological solution. *Analyst* **2021**, *146*, 2339–2347, doi:10.1039/D1AN00013F.

2. Turck, D.; Bohn, T.; Castenmiller, J.; Henauw, S. de; Hirsch-Ernst, K.I.; Maciuk, A.; Mangelsdorf, I.; McArdle, H.J.; Naska, A.; Pentieva, K.; et al. Safety of monosodium salt of l-5-methyltetrahydrofolic acid as a novel food pursuant to Regulation (EU) 2015/2283 and the bioavailability of folate from this source in the context of Directive 2002/46/EC, Regulation (EU) No 609/2013 and Regulation (EC) No 1925/2006. EFS2 2023, 21, e8417, doi:10.2903/j.efsa.2023.8417

3. www.chytre-miminko.cz/wp/wp-content/uploads/2014/09/Quatrefolic_presentation.pdf.
